# Image-based robotic-assisted conversion from partial to total knee arthroplasty under functional alignment: Comparable outcomes to primary total knee arthroplasty

**DOI:** 10.1051/sicotj/2025056

**Published:** 2026-01-22

**Authors:** Christos Koutserimpas, Enejd Veizi, Charalampos Matzaroglou, Charalampos Kontos, Sébastien Lustig, Reha Tandogan, Konstantinos Dretakis

**Affiliations:** 1 School of Rehabilitation Health Sciences, University of Patras Rio 26504 Patras Greece; 2 Department of Orthopaedic Surgery, “Hygeia” General Hospital of Athens 151 23 Athens Greece; 3 Ankara Bilkent City Hospital, Department of Orthopedics and Traumatology 06800 Ankara Turkey; 4 Univ Lyon, Claude Bernard Lyon 1 University, IFSTTAR, LBMC UMR_T9406 25 Avenue François Mitterand 69622 Lyon Turkey; 5 Orthopaedics Surgery and Sports Medicine Department, FIFA Medical Center of Excellence, Croix Rousse Hospital, Hospices Civils de Lyon, Lyon North University Hospital 103 Grande Rue de la Croix Rousse 69004 Lyon France; 6 Ortoklinik & Çankaya Hospital 06700 Ankara Turkey

**Keywords:** Functional alignment, Robotic revision, Robotic knee, Robotic unicompartmental, Robotic conversion

## Abstract

*Introduction*: Image-based robotic systems in total knee arthroplasty (TKA) allow for precise implant positioning and soft tissue balance through patient-specific preoperative planning. Functional alignment (FA) leverages the native soft tissue envelope to guide implant placement. However, its application in partial TKA conversion remains limited. This study evaluates the outcomes of image-based robotic-assisted partial-to-TKA conversion under FA principles, comparing them to a cohort of primary robotic TKAs. *Methods*: This retrospective study analyzed eight partial-to-TKA conversions performed using the image-based robotic system, with a minimum follow-up of 12 months. Demographics, implant constraints, intraoperative positioning, and postoperative outcomes were assessed. The mean age of the revision cohort was 73.3 ± 9.0 years, with a mean follow-up of 39.0 ± 11.5 months. A control group of 50 primary robotic TKAs was used for comparison. *Results*: Osteoarthritis progression (75%) and aseptic loosening (25%) were the primary reasons for revision. No stems were used, and only one patient (12.5%) required a tibial augment. Postoperative coronal alignment was 1.1° ± 1.9°, and functional outcomes (Knee Society Score-Knee: 84.5 ± 6.7, Knee Society Score-Function: 83.0 ± 7.1, Forgotten Joint Score: 72.8 ± 8.2) were comparable to the primary TKA cohort. No complications or revisions were recorded. *Conclusion*: FA-based robotic-assisted partial-to-TKA conversion yields functional and implant positioning outcomes comparable to primary robotic TKA while minimizing the need for stems, augments, or constrained implants. Further studies with larger cohorts are needed to confirm these findings. *Level of evidence*: III.

## Introduction

Robotic-assisted surgery has transformed total knee arthroplasty (TKA) by improving precision in implant positioning, enhancing alignment accuracy, and optimizing soft tissue balance [1–4]. Among the various robotic systems available, image-based robotic platforms have gained significant traction due to their ability to provide preoperative planning based on patient-specific anatomical data, while they have proven to be safe and reliable [5–7]. These systems utilize advanced imaging modalities, such as computed tomography (CT) scans, to generate detailed three-dimensional models of the knee joint, enabling surgeons to execute precise bone cuts and implant placement with real-time feedback [4, 6]. Studies have demonstrated that image-based robotic technology in primary TKA offers improved component positioning, reduced outliers in mechanical alignment, and potentially better functional outcomes compared to conventional methods [3, 6, 8–10]. However, while robotic assistance is well established in primary TKA, its application in revision procedures, particularly for converting unicompartmental knee arthroplasty (UKA) to TKA, remains relatively unexplored.

The revision of a failed UKA to a TKA presents unique challenges due to altered bony anatomy, potential bone loss, and soft tissue contracture [11]. Image-based robotic technology may offer advantages in such scenarios by enhancing surgical precision, preserving bone stock, and ensuring proper component positioning despite these complexities [12, 13]. Previous reports have described the use of imageless robotic systems in UKA-to-TKA revisions, highlighting their feasibility and satisfactory mid-term clinical outcomes [11]. However, comparative data on image-based robotic systems in this setting are limited.

The purpose of this study is to describe the surgical technique for image-based robotic-assisted UKA-to-TKA revision and to compare its outcomes to a cohort of 50 consecutive primary robotic TKAs performed under the principles of functional alignment (FA). We hypothesized that the application of image-based robotic technology in UKA-to-TKA revision would demonstrate comparable implant survivorship and functional outcomes to primary robotic TKA, while providing enhanced accuracy in component positioning and gap balancing.

## Materials and methods

This study is a retrospective analysis of a prospectively maintained database covering the period from June 2020 to April 2024. Patients included in the study had undergone conversion of a partial knee arthroplasty (UKA or patellofemoral arthroplasty) to TKA using the image-based robotic system (Mako, Stryker, Mako Surgical Corp., Fort Lauderdale, FL, USA). The minimum follow-up period was one year.

### Patient selection and data collection

Cases were identified based on the inclusion of patients with medial, lateral, or patellofemoral UKA revisions. Demographic data collected included age, sex, and body mass index (BMI). Clinical parameters analyzed included preoperative and postoperative Knee Society Score (KSS Knee and KSS Function), as well as the postoperative Forgotten Joint Score (FJS). Surgical parameters included time from UKA to revision, type of partial knee arthroplasty converted, reason for revision (e.g., osteoarthritis progression, aseptic loosening), implant constraints used, and whether intraoperative tibial recutting was required. Limb alignment was assessed pre- and postoperatively with the robotic system.

Intraoperative implant positioning was assessed using the robotic system, which provided real-time measurements of femoral and tibial coronal alignment, femoral flexion, and tibial posterior slope, all referenced to the mechanical axis of the respective bones. The rotation of the femoral component was determined in relation to the surgical trans-epicondylar axis (TEA), while the rotational alignment of the tibial component was assessed based on the Akagi line.

A total of eight patients underwent conversion from partial knee arthroplasty (UKA or patellofemoral arthroplasty) to total knee arthroplasty (TKA) using the image-based robotic system. The mean age of the study group was 73.25 years (*SD* = 9.02), and the mean total follow-up period was 39 months (*SD* = 11.46).

### Control group

The control group consisted of 50 consecutive primary total knee arthroplasties (TKAs) performed using the same image-based robotic system under the FA principles within the same study period. All patients received a cruciate retaining (CR) or posterior stabilized (PS) fixed-bearing cemented implant (Triathlon, Stryker, MI, USA). Demographic variables, including age, sex, and body mass index (BMI), were recorded for comparison with the revision cohort. Preoperative clinical assessments included the Knee Society Score (KSS Knee and KSS Function). Intraoperative data encompassed implant positioning parameters, such as femoral and tibial coronal alignment, femoral component rotation, and tibial posterior slope. Postoperative functional outcomes were evaluated using the KSS and FJS to assess differences between the primary and revision cohorts.

The control group (50 consecutive primary robotic TKA cases performed with the same robotic system during the study period) had a mean follow-up of 47.48 months (*SD* = 3.45).

### Surgical technique

**Figure 1:**
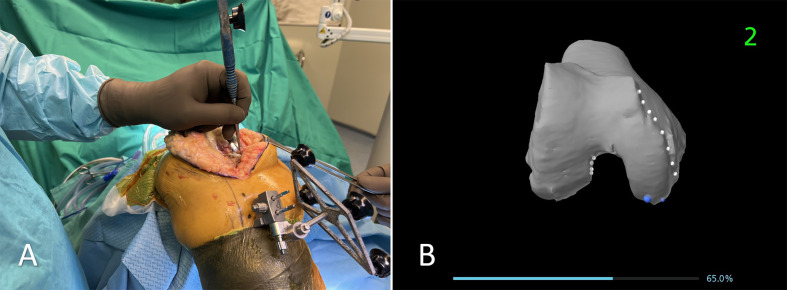
The registration is performed on the femoral component. A: Intraoperative image showing the registration process using a probe to acquire anatomical landmarks on the femoral component, ensuring accurate mapping of the bone structure. B: The corresponding visualization on the robotic system’s screen, displaying the registered points on the 3D model of the femur.

**Figure 2:**
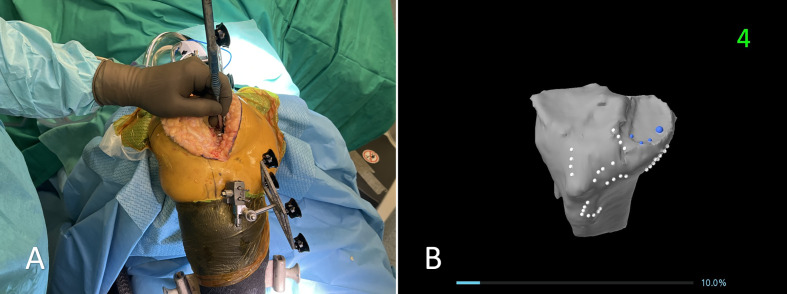
The registration is performed on the tibial component. A: Intraoperative image showing the registration process using a probe to acquire anatomical landmarks on the tibia. The original polyethylene insert has been removed prior to registration, as the preoperative CT scan does not capture it. B: The corresponding visualization on the robotic system’s screen, displaying the registered points on the 3D model of the tibia.

**Figure 3:**
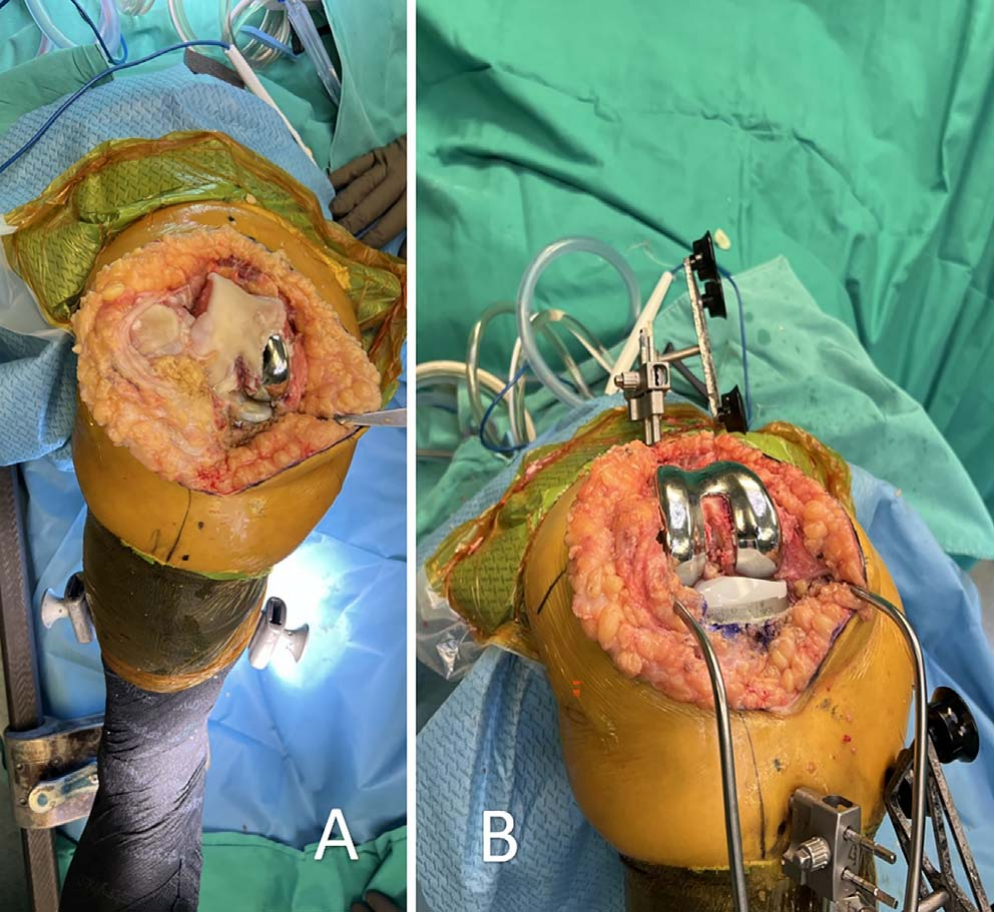
A: Intraoperative view after surgical exposure, revealing the unicompartmental knee arthroplasty implant with progressive osteoarthritis affecting the other compartment of the knee, necessitating conversion to total knee arthroplasty. B: Final implantation of a cruciate-retaining total knee prosthesis with no augments, demonstrating successful conversion while preserving the native soft tissue envelope and bone stock.

The surgical technique began with the utilization of the previous skin incision to maintain soft tissue integrity. Femoral pins were securely placed bicortically in the diaphysis, while tibial pins were inserted at a perpendicular angle to the medial surface of the tibia, approximately 45° medial to the sagittal midline [14]. Registration of the femur and tibia was performed with the existing implants in place; however, the tibial insert was removed to facilitate accurate tibial registration. (Figures 1 and 2) The procedure adhered to a “restricted” FA approach, ensuring the tibial component’s coronal positioning remained within a range of 3° varus to 2° valgus, while the femoral component was planned between 3° valgus and 3° varus. For CR designs, the posterior slope was adjusted between 0° and 3° based on patient-specific anatomy, whereas for PS designs, it was limited to 0°–1°. Although rotational alignment was not strictly constrained, precise trochlear positioning was emphasized to prevent patellofemoral mismatch. Sagittal adjustments were made to avoid anterior notching, employing increased femoral flexion or anterior translation, when necessary, with a combined flexion limit (including tibial slope and femoral flexion) of 10°. To evaluate mediolateral laxities, a trial insert was placed following tibial registration, allowing for accurate soft tissue assessment before finalizing component positioning within the predefined alignment parameters. Following this, trialing was performed to assess overall implant stability and joint kinematics. If necessary, tibial or femoral augments were used to optimize implant fit and restore joint balance. All patients received a CR or PS fixed-bearing cemented implant (Triathlon, Stryker, MI, USA). (Figure 3)

### Ethical approval

This study was carried out in accordance with the ethical standards set by both institutional and national research committees, following the principles of the 1964 Declaration of Helsinki and its later revisions or equivalent ethical guidelines. Ethical approval was granted by the Scientific Committee of Hygeia Hospital in Athens (Reference No. 663, 20/12/2023). Due to the retrospective nature of the study and the use of anonymized data, formal patient consent was not required, as per institutional regulations.

### Statistical analysis

Descriptive statistics were used to summarize demographic and surgical data. Continuous variables were reported as mean ± standard deviation (*SD*), while categorical variables were expressed as frequency and percentage. Independent t-tests were used to compare preoperative and postoperative clinical outcomes. Statistical significance was set at *p* < 0.05.

## Results

The majority of cases involved medial unicompartmental knee arthroplasty (87.5%, *n* = 7), while one patient (12.5%) had undergone patellofemoral arthroplasty. The primary reason for revision was osteoarthritis progression in the untreated compartments (75%, *n* = 6), followed by aseptic loosening of the UKA implant (25%, *n* = 2). The mean time from the initial partial knee arthroplasty to revision was 72.25 months (SD = 45.02).

Preoperatively, the mean coronal alignment measured by the robotic system indicated a varus alignment of 3.88° (*SD* = 6.13), which was corrected postoperatively to 1.13° (*SD* = 1.89). No femoral or tibial stems were used in any of the cases, and only one patient (12.5%) required tibial augments (medial augment). Additionally, one patient (12.5%) required tibial recutting. No intraoperative or postoperative complications were observed, and no patients required revision surgery during the follow-up period.

The positioning of the implants in the study group is summarized in Table 1. Femoral implant positioning demonstrated a mean valgus angle of 0.2° (*SD* = 1.28) and a mean external rotation of 1.6° (*SD* = 1.29). The femoral implant was positioned in flexion at an average of 6.2° (*SD* = 1.92). The tibial component was implanted with a mean varus angle of 0.38° (*SD* = 1.49) and a mean posterior slope of 1.6° (*SD* = 0.89).

**Table 1:** Positioning of the implants for the study group (revision from partial to total knee) under the principles of functional alignment. Positioning of the implants with reference to the femoral and tibial mechanical axes. Rotation of the femoral implant with reference to the transepicondylar axis.

Implants positioning		
Femoral implant positioning	*Valgus*	0.2 (*SD* = 1.28)
	*External rotation*	1.6 (*SD* = 1.29)
	*Flexion*	6.2 (*SD* = 1.92)
Tibial implant positioning	*Varus*	0.38 (1.49)
	*Slope*	1.6 (*SD* = 0.89)

### Comparison with the control group

The control group consisted of 50 consecutive primary robotic TKA cases performed with the same robotic system during the study period. Preoperative characteristics, including age, BMI, and functional scores, were comparable between the groups (Table 2). The study group had a higher proportion of female patients (75% vs. 48%), but this difference was not statistically significant. No revisions were recorded in this sample.

**Table 2:** Preoperative comparative data between the study (revision from partial to total knee arthroplasty) and control (primary robotic total knee arthroplasty) groups.

	Study group (*N* = 8)	Control group (*N* = 50)	*p*-value
Age	73.25 (*SD* = 9.02)	72.32 (*SD* = 7.26)	0.75
BMI	29.63 (*SD* = 3.46)	28.24 (*SD* = 4.49)	0.41
Female Gender (%)	75%	48%	0.16
KSS Function (preoperatively)	54.25 (*SD* = 21.83)	60.4 (*SD* = 19.23)	0.41
KSS Knee (preoperatively)	55.38 (*SD* = 9.21)	62.71 (*SD* = 13.39)	0.14

Postoperatively, both groups demonstrated similar functional outcomes, with no significant differences in KSS Function, KSS Knee, or FJS (Table 3). Implant constraints and insert thickness were also analyzed, with a higher proportion of inserts thicker than 9 mm in the study group (62.5% vs. 34%), though this did not reach statistical significance.

**Table 3:** Postoperative data between the study (revision from partial to total knee arthroplasty) and control (primary robotic total knee arthroplasty) groups.

	Study group (*N* = 8)	Control group (*N* = 50)	*p*-value
CR (%)	50%	46%	0.83
Insert > 9 mm (%)	62.5%	34%	0.13
KSS Function (at final follow-up)	93.13 (*SD* = 6.49)	90.28 (*SD* = 10.75)	0.47
KSS Knee (at final follow-up)	96 (*SD* = 5.55)	92 (*SD* = 9.13)	0.24
FJS	87.38 (*SD* = 10.24)	80.32 (*SD* = 23.03)	0.4

## Discussion

This study presents the surgical technique and clinical outcomes of converting a partial knee arthroplasty to a TKA using an image-based robotic system under the principles of FA. Two critical technical steps were identified: (1) removing the original tibial insert prior to tibial registration to ensure accurate mapping of the remaining bone stock; this step requires that the retained femoral component is not grossly unstable and maintains at least some soft-tissue attachments to allow for reliable registration, and (2) performing intraoperative evaluation of mediolateral laxity in both extension and flexion using a trial insert to guide soft tissue balancing in accordance with functional alignment principles. The results demonstrated that this approach allowed for precise implant positioning with minimal need for additional augments or stems, with only one patient requiring a tibial augment. Furthermore, functional outcomes and implant positioning metrics were comparable to those observed in primary robotic-assisted TKA, suggesting that image-based robotic technology can effectively facilitate UKA-to-TKA conversion without compromising stability or alignment. Notably, no intraoperative or postoperative complications were reported, and no revisions were required during the follow-up period, further supporting the feasibility and reliability of this technique.

Osteoarthritis progression (75%) and aseptic loosening (25%) were the main reasons for conversion to TKA, consistent with previous reports identifying degenerative changes in the lateral or patellofemoral compartments as the leading cause of UKA failure [15]​. Aseptic loosening is also a major cause of failure, often related to malpositioning or polyethylene wear [16]. Failures are typically multifactorial, with pain, stiffness, or instability contributing, while registry data highlight implant-related issues yielding revision rates similar to primary TKA [11, 16]​.

Limited need for PS implants, augments, stems, or larger inserts reflects the use of FA principles, which preserve the native soft tissue envelope and let intrinsic biomechanics guide implant alignment and balance [17, 18]. Under this approach, the soft tissue structures act as the “DNA” of the knee, eliminating the need for extensive ligament releases and promoting a more natural joint reconstruction [10, 18–24]. This method contrasts with conventional revision strategies that often necessitate the use of augments, stems, or constrained implants to compensate for instability or bone loss [25]. By preserving native soft tissue balance, FA allows the use of primary implants in revision cases, limiting added constraint and larger inserts. Within this framework, both CR-type inserts, such as the cruciate substituting, and PS designs have been shown to achieve comparable clinical outcomes through optimized gap balancing and alignment [9]. Thus, CR or PS selection can be based on patient anatomy and surgeon preference without affecting outcomes under FA. Our findings support the feasibility of FA-guided robotic revision TKA in achieving stable results with minimal implant modification.

Compared with primary robotic-assisted TKA under FA, partial-to-TKA conversions showed similar outcomes. Functional scores (KSS, FJS) and implant alignment were not significantly different, highlighting FA’s capacity to enable soft tissue–guided reconstruction that preserves native biomechanics, even in revision settings [26–32]. Furthermore, the use of primary implants without stems, augments, or added constraint produced constructs comparable to primary TKA, suggesting FA mitigates structural challenges in partial-to-TKA conversion. This aligns with reports that FA-based robotic TKA improves soft tissue balance and ensures balanced knees [11–13]. Historically, UKA-to-TKA revision was linked to poorer outcomes than primary TKA, leading many surgeons to prefer primary TKA to avoid revision-related complications [33]. It seems that the use of robotic systems and the FA principles may mitigate this risk.

The use of an image-based robotic system provides key advantages in evaluating and reconstructing the anterior compartment, a fundamental aspect of FA [34]. By integrating preoperative imaging, the system allows for a more precise assessment of the anterior structures, ensuring that implant positioning respects the natural femoral trochlea, while the patellar tracking can be assessed dynamically [35]. In contrast, imageless robotic systems rely solely on intraoperative landmark acquisition and dynamic gap assessments [36, 37].

Regarding the revision of UKA to TKA using an image-based system, it is important to note that the preoperative scan registers the tibial tray but not the insert, necessitating removal of the insert before mapping. A trial insert should then be used to assess mediolateral laxity in both extension and flexion. This represents a critical step in the technique, ensuring that soft tissue balance is evaluated under natural conditions.

The study has some limitations. It is limited by its small sample size, which may affect the generalizability of the findings. However, there is currently limited evidence in the literature on FA-based robotic-assisted UKA-to-TKA conversion, making this study a valuable contribution. A key strength is the comparative analysis with a primary robotic TKA cohort, which reinforces the feasibility of using FA principles in revision settings. Additionally, the retrospective design introduces potential selection bias, and future prospective studies are needed to further validate these findings.

In conclusion, this study highlights the feasibility of applying FA principles in robotic-assisted partial-to-TKA conversion, allowing for implant positioning guided by the native soft tissue envelope and minimizing the need for stems, augments, or constrained implants. Despite the small sample size, outcomes were comparable to primary robotic TKA, supporting the use of the image-based robotic system under the FA principles as a viable approach in revision settings. Further research with larger cohorts and long-term follow-up is needed to confirm its durability and benefits.

## Data Availability

Data is available upon reasonable request to the corresponding author
